# Gene Polymorphisms of Hormonal Regulators of Metabolism in Patients with Schizophrenia with Metabolic Syndrome

**DOI:** 10.3390/genes13050844

**Published:** 2022-05-08

**Authors:** Anastasiia S. Boiko, Ivan V. Pozhidaev, Diana Z. Paderina, Irina A. Mednova, Anastasya A. Goncharova, Olga Yu. Fedorenko, Elena G. Kornetova, Arkadiy V. Semke, Nikolay A. Bokhan, Anton J. M. Loonen, Svetlana A. Ivanova

**Affiliations:** 1 Mental Health Research Institute, Tomsk National Research Medical Center of the Russian Academy of Sciences, 634014 Tomsk, Russia; anastasya-iv@yandex.ru (A.S.B.); craig1408@yandex.ru (I.V.P.); osmanovadiana@mail.ru (D.Z.P.); irinka145@yandex.ru (I.A.M.); goncharanastasya@gmail.com (A.A.G.); f_o_y@mail.ru (O.Y.F.); kornetova@sibmail.com (E.G.K.); asemke@tnimc.ru (A.V.S.); nikolay.bokhan.tomsk.russia@gmail.com (N.A.B.); ivanovaniipz@gmail.com (S.A.I.); 2 Department of Psychiatry, Addictology and Psychotherapy, Siberian State Medical University, 634050 Tomsk, Russia; 3 Unit of PharmacoTherapy, -Epidemiology, and -Economics, Groningen Research Institute of Pharmacy, Faculty of Science and Engineering, University of Groningen, 9713AV Groningen, The Netherlands

**Keywords:** metabolic syndrome, genetic polymorphism, *INSIG2*, *GHRL*, *LEP*, *LEPR*

## Abstract

Background: Metabolic syndrome (MetS) is a common complication of long-term treatment of persons with schizophrenia taking (atypical) antipsychotics. In this study, we investigated the existence of an association with polymorphisms of genes for four hormones that regulate energy metabolism. Methods: We recruited 517 clinically admitted white patients (269M/248F) with a verified diagnosis of schizophrenia (ICD-10) and with a stable physical condition. Participants were classified for having or not having MetS and genotyped for 20 single-nucleotide polymorphisms (SNPs) in the genes encoding *insulin-induced gene 2* (*INSIG2*), *ghrelin* (*GHRL*), *leptin* (*LEP*), and *leptin receptor* (*LEPR*). Results: The 139 patients (26.9%) with MetS were significantly more likely to be women, older, and ill longer, and had a larger body mass index (BMI). Four polymorphisms (rs10490624, rs17587100, rs9308762, and rs10490816) did not meet the Hardy–Weinberg equilibrium (HWE) criterion and were excluded. Only genotypes and alleles of the rs3828942 of *LEP* gene (chi2 = 7.665, *p* = 0.022; chi2 = 5.136, *p* = 0.023) and the genotypes of the rs17047718 of *INSIG2* gene (chi2 = 7.7, *p* = 0.021) had a significant association with MetS. Conclusions: The results of our study suggest that the *LEP* and *INSIG2* genes play a certain causal role in the development of MetS in patients with schizophrenia.

## 1. Introduction

Antipsychotics are the main pharmacological agents used to treat schizophrenia patients, but long-term use of most of these preparations increases the risk of developing metabolic syndrome (MetS) [1,2,3]. MetS has associations with cardiovascular risk factors, which increases the risk of death in patients with mental disorders. The hormonal regulators of metabolism and their encoding genes may provide information about the mechanism and be potential biomarkers for susceptibility to the development of MetS [4,5].

Previous studies have suggested some candidate genes for MetS, including the *insulin-induced gene 2* (*INSIG2*) [6,7], *ghrelin* gene (*GHRL*) [8,9,10,11,12,13,14], *leptin* (*LEP*) [7,11,15,16,17,18,19], and *leptin receptor* genes (*LEPR*) [11,16,17,19,20]. For instance, *INSIG2* rs17047764 was shown to be associated with antipsychotic-related weight gain [6], whereas rs17587100, rs10490624, and rs17047764 localized within or near the *INSIG2* gene had a strong association with clozapine-induced BMI gain in patients with schizophrenia [7]. *GHRL* rs27647, rs26802, and rs696217 are associated with certain components of MetS in elderly people [10].

In a previous study, we have measured serum levels of the adipokines leptin, adiponectin, tumor necrosis factor-α, and interleukin-6, and related them to the existence of (components of) MetS in people with schizophrenia [21]. The results suggest that leptin in particular is elevated in people with MetS. We think it is interesting to investigate whether this also has a genetic component.

*Insulin-induced gene 2* (*INSIG2*) encodes for an Insig2 protein of 225 amino acids that plays an important role in inhibiting the synthesis of cholesterol and other lipids in adipocytes [22,23]. By binding to the sterol regulatory element-binding protein (SREBP) and the cleavage-activating protein (SCAP), Insig prevents this SCAP/SREBP complex from leaving the site of synthesis in the endoplasmic reticulum and coming into action. Among other things, Insig prevents the transcription of 3-hydroxy-3-methylglutaryl-coenzyme A reductase (HMG-CoA reductase) and also promotes the sterol-dependent degradation of HMG-CoA reductase. In this way, Insig2 is assigned an important regulatory role in adipocyte differentiation, cholesterol homeostasis, lipogenesis, and glucose metabolism [22].

Ghrelin is a peptide hormone of 28 amino acids that is produced primarily in the stomach and was first described in 1999 [24,25]. It is secreted in two forms: if less than 10% is acylated and if it has affinity for the growth hormone secretagogue receptor 1a (GHSR1a). The remainder is not acylated and reacts with a yet unknown receptor. Ghrelin has a multitude of biological effects, the best known of which is the promotion of appetite, whereby acting on the hypothalamic arcuate nucleus (ARC) is thought to be the main mechanism [25]. In addition to regulating this homeostatic food intake, peripherally secreted ghrelin is also involved in hedonic-motivated craving for food [24]. Moreover, ghrelin, in combination with leptin and orexin, has been attributed a role in the development of obesity in people with short sleep duration [26]. A meta-analysis of 21 studies with 2250 participants found that short sleep duration was associated with increased ghrelin levels, while sleep deprivation had a significant effect on the levels of both leptin and ghrelin [27]. This is related to the fact that food intake also follows a circadian rhythm, and that ghrelin and leptin play an important role in the circadian regulation of homeostatic feeding [28].

The adipokine leptin is mainly produced by mature adipocytes of the white adipose tissue and is one of the major hormonal signaling mediators from adipose tissue to other tissues, including the central nervous system [29,30,31,32]. It was discovered in 1994 after cloning the mouse *obese* gene [33]. The transcript of the human *LEP* gene is a polypeptide of 167 amino acids [30,32], but it is secreted into the blood in an active form of 146 amino acids [34]. Leptin achieves biological effects by binding to a leptin receptor, of which at least six isoforms exist by alternative splicing or posttranslational modification of the same gene transcript [29]. One of these, the LepRe isoform, is not membrane-bound, and acts as the major transport protein in the blood stream and as an inhibitor of transport through the blood–brain barrier [30,32]. Among the others, the long LepRb isoform is the most important and is found at various sites in the central nervous system and in tissues beyond [29]. Binding of leptin to this receptor results in the recruitment and activation of Janus kinase 2 (JAK2) and, ultimately, in the activation of the signal transducer and activator of transcription 3 (STAT3) signaling [30,32]. Although leptin can also reach the nucleus arcuatus in the hypothalamus by other routes [30], it must cross the blood–brain barrier (BBB) in order to reach the leptin receptors. Although still controversial, it has been postulated that the short LepRa isoform performs this function as a leptin transporter across the BBB, while the long LepRb isoform acts as the receptor proper [35,36]. An important aspect of diet-induced obesity is the occurrence of resistance to the effects of exogenous leptin and also ghrelin [37,38]. The mechanism of this and the role it may play in the development of obesity are not entirely clear [38].

Most review articles take a deep look at how these hormones achieve their effects via the hypothalamus and brainstem [30,39]. In doing so, in our opinion, this part of the complex emotional response is not sufficiently framed within the larger picture of the complete emotional behavioral response [40]. We believe that the hypothalamus and brainstem play primarily a role in the mutual adjustment of various “visceral” body functions to momentary needs and by monitoring the balance between them. In this case, this means that the hormones described primarily influence “homeostatic” energy regulation via the hypothalamus. This does not exclude their involvement in the development of weight gain and MetS, but makes this involvement more modest, since behavior regulated in other ways will also have much influence. Because this involves a close interplay of factors in the fine regulation of energy homeostasis, genetic changes in the sensitivity of receptors may nevertheless be additional causes of derailment that can manifest themselves in the development of MetS. For this reason, studying the relationship between polymorphisms of *INSIG2*, *GHRL*, *LEP*, and *LEPR* was a logical step.

## 2. Materials and Methods

### 2.1. Patients

The study complied with the Declaration of Helsinki (1975, revised in Fortaleza, Brazil, 2013) and the protocol was approved by the local Bioethical Committee. We recruited 517 patients with schizophrenia from different psychiatric clinics of the Siberian region in the Russian Federation. The detailed characteristics of the studied group are presented in Boiko et al. (2021) [41]. The informed consent of all participants was obtained.

The main criteria for the inclusion of patients in the study were a diagnosis of schizophrenia according to the International Classification of Diseases, 10th Revision (ICD-10) criteria, as assessed by applying a structured clinical interview (Structured Clinical Interview for the DSM [SCID]); age 18–65 years; white appearance; and the absence of severe organic pathology or somatic disorders in the stage of decompensation.

The antipsychotic and concomitant therapy received at the time of the examination (drugs, dosages used, duration of current drug use) was assessed, as well as previous antipsychotic and concomitant somatic therapy during the preceding six months. We used the chlorpromazine equivalent (CPZeq) daily dosage to standardize the dose, efficacy, and side effects of antipsychotics [42].

MetS was diagnosed according to the criteria of the International Diabetes Federation (IDF, 2005) [43]. The differences between total cholesterol, high-density lipoproteins, triglyceride, and glucose in the groups with and without MetS are presented in Supplementary Table S1.

### 2.2. Blood Sampling

To obtain blood serum, vacutainer tubes containing SiO_2_ as a coagulation activator (CAT) were used. We applied the standard procedure in which blood samples were centrifuged at 2000× *g* at 4 °C for 30 min, followed by storage of the serum aliquots at −20 °C (or −80 °C) until analysis. For DNA isolation, vacutainer tubes containing EDTA were used, and the standard phenol–chloroform method was applied.

### 2.3. Biochemical Parameters

Total cholesterol, high-density lipoproteins, triglyceride, and glucose were measured by commercial colorimetric enzymatic methods (Cormay, Poland).

### 2.4. Genetic Analysis

Genotyping of 20 single-nucleotide polymorphisms (SNPs) in the *ghrelin* gene (*GHRL*: rs10490816, rs26312, rs26802, rs27647, rs35682, rs35683), *leptin* gene (*LEP*: rs2167270, rs3828942, rs10954173, rs4731426), *leptin receptor gene* (*LEPR*: rs1805094, rs1137101, rs1171276, rs1805134), and *insulin-induced gene 2* (*INSIG2*: rs10490624, rs12623648, rs17047718, rs17587100, rs9308762, rs17047731) was carried out using a MassARRAY Analyzer 4 mass spectrometer (Agena Bioscience) and a QuantStudio 3D Digital PCR System Life Technologies amplifier (Applied Biosystems) using TaqMan Validated SNP Genotyping Assay kits (Applied Biosystems), based at The Core Facility “Medical Genomics” (Tomsk National Research Medical Center of the Russian Academy of Sciences). Selection criteria of the studied SNPs included a citation in the previous genetic studies of MetS components as described in the introduction, and a minor allele frequency (MAF) of > 5%. Basic information on these SNPs is described in Supplementary Table S2.

### 2.5. Statistical Analysis

Statistical analysis was carried out with SPSS software (release 23.0) and software R version 4.0.4 using standard functions, as well as additional packages “SNPassoc”, “psych”, and “dplyr”. The chi-square test was used to compare gender between the groups and the Mann–Whitney U test to compare the other demographic and clinical variables. The Hardy–Weinberg equilibrium (HWE) of genotypic frequencies was tested by the chi-square test. Pearson’s chi-squared test was used for between-group comparisons of genotypic and allelic frequencies. An assessment of the association of genotypes and alleles of the studied polymorphic variants of genes with a pathological phenotype was carried out using the odds ratio (OR) with a 95% confidence interval for the odds ratio (95% CI). The critical significance level was 0.05.

## 3. Results

A total of 517 patients receiving long-term antipsychotic therapy were examined. MetS was diagnosed in 139 patients (26.9%). Table 1 presents the main demographic and clinical parameters of the studied patient groups. The same parameters of the studied patient groups depending on gender are presented in Supplementary Table S3.

In our sample, MetS was more often diagnosed in women with schizophrenia. The patients with MetS were significantly older (*p* < 0.0001), and the duration of illness in these patients was significantly longer than that in patients without MetS (*p* = 0.001). The study groups also showed significant differences in body mass index (*p* < 0.0001).

Checking the frequency distribution of genotypes in the study group of patients showed that the frequency distribution corresponds to the Hardy–Weinberg equilibrium except for polymorphisms rs10490624 (*LEP*), rs17587100 and rs9308762 (*INSIG2*), and rs10490816 (*GHRL*) (Supplementary Table S2). These polymorphisms were excluded from further analysis.

Statistical analysis of *LEP* and *INSIG2* in the group of schizophrenia patients revealed a significant association of genotypes and alleles of the rs3828942 of *LEP* gene (chi2 = 7.665, *p* = 0.022; chi2 = 5.136, *p* = 0.023) (Table 2) and the genotypes of the rs17047718 of *INSIG2* gene (chi2 = 7.7, *p* = 0.021) with MetS (Table 3). The genotype AA and the allele A of the rs3828942 have a predisposing effect on the development of MetS (OR1 = 2.06, 95%CIs: 1.16–3.64; OR2 = 1.4, 95%CIs: 1.05–1.87). For the polymorphisms of the *GHRL* and *LEPR* genes, no associations were found with MetS in the study group of patients (Table 4 and Table 5). 

## 4. Discussion

In this study, with a naturalistic experimental design, we genotyped a group of 517 physically stable patients with schizophrenia (248 women and 269 men) for 20 polymorphisms in the genes encoding insulin-induced gene 2 (*INSIG2*), ghrelin (*GHRL*), leptin (*LEP*), and the leptin receptor (*LEPR*), and examined whether differences existed between 378 patients without and 139 with MetS. We found a significant relationship with the occurrence of MetS only for rs3828942 of the *LEP* gene and rs17047718 of the *INSIG2* gene.

In our population, MetS is significantly more common in women than in men. This may be sincerely so, but can also be related to the differences in the IDF (2005) criteria for men and women. The significant differences between patients with and without MetS, in terms of age and duration of illness, are probably related to longer-term treatment with (atypical) antipsychotics. The significant difference in BMI is probably causally related to having MetS.

The polymorphism rs3828942 corresponds to a variation in the intron domain of the *LEP* gene. This polymorphism has nevertheless been repeatedly shown to be associated with clinical CNS-related phenomena [44,45,46,47]. Therefore, it may be shown that this polymorphism itself, or a polymorphism with rs3828942 in equilibrium, has functional consequences. These are not necessary related to homeostatic energy regulation because this variant is probably also involved in sleep parameters [45] and anxiety [47]. Interestingly, Salerno and colleagues examined the association of leptin levels and LEP rs3828942 with generalized anxiety disorder (GAD), taking into account gender differences [47]. They showed an interaction between this genotype and the diagnosis of GAD based on leptin levels, but only in the male group [*p* = 0.0139]. Leptin was associated in a sex-dependent manner with the neurobiology of anxiety disorders because female carriers of the A allele of LEP rs3828942 showed a higher risk for GAD, while leptin levels seemed to be lower in men with GAD who were carriers of the A allele [48]. Based on these data, we can speculate that the rs3828942 may have an influence on serum leptin levels, which are involved in the pathogenesis of the MetS.

The polymorphism rs17047718 is a variant in the promoter domain of the *INSIG2* gene. Talbert and colleagues’ study [48] of quantitative adiposity and glucose homeostasis traits in 1425 Hispanics of the Insulin Resistance Atherosclerosis Family Study found a clear association of this SNP with direct computed tomography (CT)-measured adiposity phenotypes and with glucose homeostasis traits. The most prominent association was observed between rs17047718 and visceral adipose tissue (VAT), subcutaneous adipose tissue (SAT), and VAT to SAT ratio (VSR) (*p* values ranged from 0.007 to 0.044) [48]. For rs17047731, we still found a statistical trend; however, for rs12623648 and rs9308762, we found no association in our study. In their meta-analysis, Zhang et al. [6] found some association between rs17047764 and antipsychotic-related weight gain, but we did not examine this SNP. We would like to tentatively conclude that the *INSIG2* gene is probably involved in the development of MetS in people with schizophrenia treated with antipsychotic drugs.

### Limitations

Our research has several limitations. First, we studied a natural cohort and examined it trans-sectionally. It would be better to prospectively follow the occurrence of MetS in individuals randomized to certain genotypes of the SNPs studied. However, this is not feasible. This limitation means that our results are only indicative, but no less interesting. A second limitation is that our patients may have comorbidities and may use comedications, which may also influence the prevalence of MetS. We excluded patients with severe organic pathology or somatic disorders in a decompensation stage from participation, and we expect that the comedication used will be distributed at random across the genotypes, thus limiting the ultimate influence. Compared with studies conducted by cardiovascular interested scientists, the size of the population studied is modest. The reader should not lose sight of the fact that we are primarily interested in mechanistic research and that our population consists of people with schizophrenia who are stable on antipsychotics. For this reason, we also do not consider it appropriate to correct for multiple testing, but we are aware that our results do not have sufficient probative value to justify use as a biomarker.

## 5. Conclusions

The results of our study show that the *LEP* and *INSIG2* genes can play an important role in the development of MetS in patients with schizophrenia. Further study of the molecular genetic factors of MetS and the mechanisms by which antipsychotics affect metabolic parameters is necessary in order to assess the risk of metabolic disorders and the implementation and individual approach to therapeutic tactics.

## Figures and Tables

**Table 1:** Demographic and clinical parameters of the studied patient groups.

Parameter		Patients without MetS, n = 378 (73.1%)	Patients with MetS, n = 139 (26.9%)	*p* Value
Gender	Women	165 (43.7%)	83 (59.7%)	0.002
	Men	213 (56.3%)	56 (40.3%)	
Mean age (M ± SD)		39.03 ± 11.65	44.19 ± 11.51	<0.0001
Mean duration of illness (Me [Q1; Q3])		12.0 [6.0; 20.0]	17.0 [9.5; 22.5]	0.001
Mean CPZeq, dose (Me [Q1; Q3])		442.4 [250.0; 758.7]	442.4 [225.0; 778.7]	0.775
Body mass index (BMI) (M ± SD)		24.40 ± 4.85	31.04 ± 5.78	<0.0001

**Note:** Comparisons between groups have been made using the chi-squared test for gender and the Mann–Whitney U-test for the other variables. **Abbreviations**: Me [Q1; Q3]—median and quartiles (first and third); MetS: metabolic syndrome; CPZeq: chlorpromazine equivalent; M ± SD—mean and standard deviation.

**Table 2:** Distribution of alleles and genotypes of *LEP* polymorphisms in groups of patients.

SNP	Genotypes/ Alleles	Patients without MetS n (%)	Patients with MetS n (%)	OR	95%CI	χ^2^	*p*
rs2167270	G/G	111 (32.1)	51 (40.8)	1.46	0.96–2.23	4.194	0.123
	G/A	174 (50.3)	55 (44.0)	0.69	0.44–1.08		
	A/A	61 (17.6)	19 (15.2)	0.68	0.37–1.25		
	G	0.572	0.628	1.26	0.94–1.70	2.354	0.125
	A	0.428	0.372	0.79	0.59–1.07		
**rs3828942**	G/G	107 (30.9)	34 (27.2)	**0.83**	**0.53–1.32**	**7.665**	0.022 *
	G/A	184 (53.2)	55 (44.0)	**0.94**	**0.58–1.53**		
	**A/A**	55 (15.9)	36 (28.8)	**2.06**	**1.16–3.64**		
	**G**	0.575	0.492	**0.72**	**0.54–0.96**	**5.136**	0.023 *
	**A**	0.425	0.508	**1.40**	**1.05–1.87**		
rs10954173	G/G	112 (32.5)	52 (41.9)	1.50	0.99–2.29	4.199	0.123
	A/G	171 (49.6)	52 (41.9)	0.65	0.42–1.03		
	A/A	62 (18.0)	20 (16.1)	0.69	0.38–1.27		
	G	0.572	0.629	1.27	0.94–1.71	2.409	0.121
	A	0.428	0.371	0.79	0.59–1.06		
rs4731426	C/C	85 (24.6)	41 (33.1)	1.51	0.97–2.36	4.425	0.109
	G/C	179 (51.9)	58 (46.8)	0.67	0.42–1.08		
	G/G	81 (23.5)	25 (20.2)	0.64	0.36–1.15		
	C	0.506	0.565	1.27	0.95–1.70	2.521	0.112
	G	0.494	0.435	0.79	0.59–1.06		

In bold and with *: Significant difference *p* < 0.05.

**Table 3:** Distribution of alleles and genotypes of *INSIG2* polymorphisms in groups of patients.

SNP	Genotypes/ Alleles	Patients without MetS n (%)	Patients with MetS n (%)	OR	95%CI	χ^2^	*p*
rs10490624	T/T	223 (81.7)	79 (78.2)	0.81	0.46–1.41	1.406	0.495
	C/T	27 (9.9)	12 (11.9)	1.25	0.61–2.60		
	C/C	23 (8.4)	10 (9.9)	1.23	0.56–2.69		
	T	0.866	0.842	0.82	0.52–1.29	0.746	0.388
	C	0.134	0.158	1.22	0.78–1.91		
rs12623648	G/G	317 (91.6)	113 (90.4)	0.86	0.43–1.75	2.255	0.324
	G/T	29 (8.4)	11 (8.8)	1.06	0.51–2.20		
	T/T	0 (0.0)	1 (0.8)	-	-		
	G	0.958	0.948	0.80	0.41–1.56	0.439	0.508
	T	0.042	0.052	1.25	0.64–2.45		
**rs17047718**	A/A	307 (89.0)	110 (89.4)	1.05	**0.54–2.04**	**7.700**	0.021 *
	A/G	38 (11.0)	10 (8.1)	0.73	**0.35–1.52**		
	G/G	0 (0.0)	3 (2.4)	-	-		
	A	0.945	0.935	0.84	0.46–1.53	0.331	0.565
	G	0.055	0.065	1.19	0.65–2.18		
rs17587100	A/A	237 (73.6)	77 (68.8)	0.79	0.49–1.26	1.687	0.430
	C/A	73 (22.7)	31 (27.7)	1.31	0.80–2.14		
	C/C	12 (3.7)	4 (3.6)	1.03	0.32–3.27		
	A	0.849	0.826	0.84	0.56–1.26	0.694	0.405
	C	0.151	0.174	1.19	0.79–1.79		
rs9308762	T/T	189 (54.9)	55 (44.4)	0.65	0.43–0.99	4.116	0.128
	C/T	99 (28.8)	47 (37.9)	1.63	1.03–2.58		
	C/C	56 (16.3)	22 (17.7)	1.35	0.76–2.40		
	T	0.693	0.633	0.76	0.56–1.04	3.028	0.082
	C	0.307	0.367	1.31	0.97–1.78		
rs17047731	G/G	305 (88.4)	110 (88.7)	1.03	0.54–1.97	4.945	0.084
	G/A	40 (11.6)	12 (9.7)	0.83	0.42–1.64		
	A/A	0 (0.0)	2 (1.6)	-	-		
	G	0.942	0.935	0.89	0.49–1.62	0.139	0.709
	A	0.058	0.065	1.12	0.62–2.04		

In bold and with *: Significant difference *p* < 0.05.

**Table 4:** Distribution of alleles and genotypes of *LEPR* polymorphisms in groups of patients.

SNP	Genotypes/ Alleles	Patients without MetS n (%)	Patients with MetS n (%)	OR	95%CI	χ^2^	*p*
rs1805094	G/G	256 (74.0)	95 (76.0)	1.11	0.69–1.79	0.470	0.790
	C/G	84 (24.3)	27 (21.6)	0.87	0.53–1.42		
	C/C	6 (1.7)	3 (2.4)	1.35	0.33–5.50		
	G	0.861	0.868	1.06	0.69–1.62	0.070	0.791
	C	0.139	0.132	0.94	0.62–1.44		
rs1137101	G/G	95 (27.5)	35 (28.2)	1.03	0.66–1.63	0.453	0.797
	G/A	170 (49.3)	57 (46.0)	0.91	0.56–1.49		
	A/A	80 (23.2)	32 (25.8)	1.09	0.62–1.91		
	G	0.522	0.512	0.96	0.72–1.29	0.068	0.794
	A	0.478	0.488	1.04	0.78–1.39		
rs1171276	A/A	240 (69.6)	91 (73.4)	1.21	0.76–1.91	0.614	0.736
	A/G	101 (29.3)	29 (23.4)	0.76	0.47–1.22		
	G/G	4 (1.2)	4 (3.2)	2.64	0.65–10.77		
	A	0.842	0.851	1.07	0.71–1.60	0.107	0.744
	G	0.158	0.149	0.93	0.62–1.40		
rs1805134	T/T	228 (66.1)	86 (69.4)	1.16	0.75–1.81	1.008	0.604
	C/T	104 (30.1)	33 (26.6)	0.84	0.53–1.34		
	C/C	13 (3.8)	5 (4.0)	1.02	0.35–2.95		
	T	0.812	0.827	1.11	0.76–1.62	0.274	0.601
	C	0.188	0.173	0.90	0.62–1.32		

**Table 5:** Distribution of alleles and genotypes of *GHRL* polymorphisms in groups of patients.

SNP	Genotypes/ Alleles	Patients without MetS n (%)	Patients with MetS n (%)	OR	95%CI	χ^2^	*p*
rs10490816	G/G	170 (49.1)	58 (46.4)	0.90	0.59–1.35	1.05	0.592
	G/C	133 (38.4)	50 (40.0)	1.10	0.71–1.71		
	C/C	43 (12.4)	17 (13.6)	1.16	0.61–2.19		
	G	0.684	0.664	0.91	0.67–1.24	0.321	0.571
	C	0.316	0.336	1.09	0.80–1.49		
rs26312	G/G	283 (82.0)	101 (80.8)	0.92	0.55–1.56	0.34	0.844
	A/G	58 (16.8)	23 (18.4)	1.11	0.65–1.89		
	A/A	4 (1.2)	1 (0.8)	0.70	0.08–6.34		
	G	0.904	0.900	0.95	0.59–1.55	0.040	0.842
	A	0.096	0.100	1.05	0.65–1.71		
rs26802	T/T	153 (44.2)	61 (48.8)	1.20	0.80–1.81	1.676	0.433
	G/T	154 (44.5)	51 (40.8)	0.83	0.54–1.28		
	G/G	39 (11.3)	13 (10.4)	0.84	0.42–1.67		
	T	0.665	0.692	1.13	0.83–1.55	0.619	0.431
	G	0.335	0.308	0.88	0.65–1.20		
rs27647	T/T	128 (37.0)	44 (35.2)	0.93	0.60–1.42	0.743	0.690
	C/T	169 (48.8)	62 (49.6)	1.07	0.68–1.67		
	C/C	49 (14.2)	19 (15.2)	1.13	0.60–2.12		
	T	0.614	0.600	0.94	0.70–1.27	0.155	0.694
	C	0.386	0.400	1.06	0.79–1.43		
rs35682	A/A	93 (26.9)	34 (27.2)	1.02	0.64–1.61	1.197	0.550
	G/A	178 (51.4)	58 (46.4)	0.89	0.54–1.46		
	G/G	75 (21.7)	33 (26.4)	1.20	0.68–2.12		
	A	0.526	0.504	0.92	0.69–1.22	0.357	0.550
	G	0.474	0.496	1.09	0.82–1.46		
rs35683	C/C	96 (27.7)	34 (27.2)	0.97	0.61–1.54	1.482	0.477
	C/A	175 (50.6)	58 (46.4)	0.94	0.57–1.53		
	A/A	75 (21.7)	33 (26.4)	1.24	0.71–2.19		
	C	0.530	0.504	0.90	0.67–1.20	0.511	0.475
	A	0.470	0.496	1.11	0.83–1.48		

## Data Availability

The datasets generated for this study will not be made publicly available, but they are available on reasonable request to Svetlana A. Ivanova (ivanovaniipz@gmail.com), following approval of the Board of Directors of the MHRI, in line with local guidelines and regulations.

## Supplementary Materials

The following supporting information can be downloaded at: https://www.mdpi.com/article/10.3390/genes13050844/s1: Table S1: The concentration of glucose and lipids in the blood serum of the studied patient groups. Table S2: The basic information of analyzed polymorphic variants. Table S3: Demographic and clinical parameters of the studied patient groups depending on gender.
