# Supplementary material for: Gene Polymorphisms of Hormonal Regulators of Metabolism in Patients with Schizophrenia with Metabolic Syndrome
Source: Genes (Basel). 2022 May 8;13(5):844. doi: 10.3390/genes13050844 (PMC9141866; doi:10.3390/genes13050844)
Supplement: Supplementary file 1 [file genes-13-00844-s001.zip › genes-1697237-supplementary.pdf]

Table S1. The concentration of glucose and lipids in the blood serum of the studied patient groups.

Parameter, mmol/L	Patients without MetS	Patients with MetS	*p* value
Glucose	4,9 [4,3; 5,4]	5,3 [4,7; 6,0]	<0.0001
Total cholesterol	4,45 [3,85; 5,00]	4,80 [4,10; 5,57]	0.001
High-density lipoproteins	1,08 [0,86; 1,32]	0,86 [0,70; 1,06]	<0.0001
Triglyceride	1,14 [0,81; 1,48]	2,03 [1,60; 2,50]	<0.0001

Comparisons between groups were performed using Mann–Whitney U-test.

Table S2. The basic information of analyzed polymorphic variants.

Gene	Polymorphic variant	Chromosome: Location	Region	HWE χ2	HWE p
LEPR	rs1805094	1:65610269	missense variant	0.000	1.000
	rs1137101	1:65592830	missense variant	0.059	0.808
	rs1171276	1:65521766	intron variant	0.556	0.456
	rs1805134	1:65601426	synonymous variant	0.622	0.430
LEP	rs2167270	7:128241296	5 prime UTR variant	0.248	0.618
	rs3828942	7:128254252	intron variant	0.664	0.415
	rs10954173	7:128251387	intron variant	0.062	0.803
	rs4731426	7:128242017	intron variant	1.113	0.291
INSIG2	rs10490624	2:118104916	intron variant	**99.085**	**<0.001**
	rs12623648	2:118112002	regulatory region variant	0.000	1.000
	rs17047718	2:118079736	intergenic variant	0.116	0.734
	rs17587100	2:118080834	regulatory region variant	**4.002**	**0.045**
	rs9308762	2:118106298	intron variant	**54.418**	**<0.001**
	rs17047731	2:118083157	intergenic variant	0.000	1.000
GHRL	rs10490816	3:10293488	non coding transcript exon variant	**4.577**	**0.032**
	rs26312	3:10291174	5 prime UTR variant	0.046	0.830
	rs26802	3:10290681	intron variant	0.074	0.786
	rs27647	3:10290784	5 prime UTR variant	0.468	0.494
	rs35682	3:10287098	non coding transcript exon variant	0.165	0.685
	rs35683	3:10286566	non coding transcript exon variant	0.026	0.871

Note: HWE χ2 и HWE p – chi-square and p-value statistics to test the frequency distribution according to the Hardy-Weinberg equilibrium.

Table S3. Demographic and clinical parameters of the studied patient groups depending on gender.

Parameter	Men			Women		
	**Patients without MetS**	**Patients with MetS**	***p* value**	**Patients without MetS**	**Patients with MetS**	***p* value**
Mean age (M ± SD)	38.51 ± 11.62	39.20 ± 11.26	0.694	40.21 ± 12.01	47.54 ± 10.75	<0.0001
Mean duration of illness (Me [Q1; Q3])	13.0 [7.0; 21.0]	15.0 [5.0; 22.0]	0.947	11.0 [5.0; 17.0]	19.0 [11.0; 25.0]	<0.0001
Mean CPZeq, dose (Me [Q1; Q3])	482.5 [250.0; 775.0]	426.5 [215.7; 643.8]	0.508	339.9 [200.0; 750.0]	417.4 [225.0; 765.0]	0.214
Body mass index (BMI) (M ± SD)	23.39 ± 3.36	30.65 ± 4.08	<0.0001	25.63 ± 6.06	31.32 ± 6.76	<0.0001
